# Economic evaluation of sildenafil for the treatment of pulmonary arterial hypertension in Indonesia

**DOI:** 10.1186/s12913-019-4422-5

**Published:** 2019-08-14

**Authors:** Oktavia Lilyasari, Yusuf Subekti, Nur Atika, Lucia Kris Dinarti, Septiara Putri, Cicih Opitasari, Anggita Bunga Anggraini, Thanaporn Bussabawalai, Yot Teerawattananon

**Affiliations:** 1 Department of Cardiology, Medical School, University of Indonesia - Harapan Kita National Hospital, Jakarta, Indonesia; 2 Indonesian Health Technology Assessment Committee, Jakarta, Indonesia; 3 Department of Cardiology, Gadjah Mada University, Jogyakarta, Indonesia; 4 Faculty of Public Health, University of Indonesia, Jakarta, Indonesia; 5 National Institute of Health Research, Ministry of Health, Jakarta, Indonesia; 6 Health Intervention and Technology Assessment Program (HITAP), Ministry of Public Health, Nonthaburi, Thailand

**Keywords:** Economic evaluation, Sildenafil, Beraprost, Pulmonary arterial hypertension, PAH, Indonesia

## Abstract

**Background:**

This study aims to assess the cost-effectiveness and budget impact of adopting sildenafil to the benefits package for the indication of pulmonary arterial hypertension (PAH), compared to beraprost.

**Methods:**

Based on a societal perspective, a model-based economic evaluation was performed using local and international data to quantify the potential costs and health-related outcomes in terms of life years (LYs) and quality-adjusted life years (QALYs).

**Results:**

The economic model calculated the incremental cost-effectiveness ratio (ICER) per QALY gained for using sildenafil as first-line therapy compared to beraprost for the patient in functional class (FC) II and III, i.e. USD 3098 and USD 2827, respectively. The results indicated that in spite of sildenafil being more expensive than beraprost, generic sildenafil could potentially be a good value for money since ICER per QALY is below one times gross domestic product (GDP) per capita in Indonesia. Furthermore, budget impact analysis estimated that the incremental budget needed within 5 years for including sildenafil compared to beraprost for PAH patients starting in FC II and FC III was USD 436,775 and USD 3.6 million, respectively.

**Conclusions:**

Compared to beraprost, sildenafil would be preferable for the treatment of PAH patients in FC II and FC III in Indonesia. The additional budget for adopting sildenafil compared to beraprost as the treatment of PAH in the benefits package was estimated at around USD 4.0 million.

**Electronic supplementary material:**

The online version of this article (10.1186/s12913-019-4422-5) contains supplementary material, which is available to authorized users.

## Background

Pulmonary arterial hypertension (PAH) is a disease characterized by a mean pulmonary artery pressure of more than 25 mmHg at rest [1–3]. In normal conditions, mean pulmonary artery pressure is usually between 8 and 20 mmHg [4]. PAH is a progressive disease, ultimately leading to right heart failure and death [5]. The etiologies of PAH are unknown or idiopathic, familial, or associated with certain diseases, such as congenital heart disease, connective tissue disease, portal hypertension, and HIV infection, or exposure to toxins and drugs including appetite suppressant drugs [6].

The World Health Organization (WHO) classifies PAH according to symptoms experienced by patients, including shortness of breath, fatigue, chest pain, syncope, and limitation of physical activities. PAH is grouped into four functional classifications (FC) based on disease severity, ranging from FC I to FC IV, in order of increasing severity. Functional classification is an important factor that must be considered in determining the degree of severity (regardless of the cause), the target of treatment during follow-up, and predictors of patient survival assessment [7, 8].

Establishing a PAH diagnosis is problematic because patients are usually unaware of the disease symptoms, making it difficult to estimate the number of PAH cases [1]. Nevertheless, some statistics are available from developed countries; for example, in France, the incidence and prevalence of PAH is approximately 2.4 cases and 15 cases, respectively, per one million adults per year [9]. In Scotland, the incidence and prevalence of PAH is 7.6 cases and 26 cases, respectively, per one million adults per year. In Indonesia, however, the prevalence is still unclear.

Currently, no treatment is available to cure PAH completely. However, providing treatment in the early FC would provide a better result than in late FC [7] and several treatments may improve the patient’s quality of life (QOL) [4]. According to European guidelines, the supportive therapies include anticoagulants, diuretics, oxygen, and digoxin [1]. Drugs, such as sildenafil, inhaled iloprost, bosentan, and beraprost can reduce pulmonary artery pressure, which would improve the patient’s quality of life and survival [10]. In Indonesia, beraprost and iloprost are the two available PAH drugs on the market, but only beraprost is listed in the National Formulary. Bosentan is not accessible in Indonesia, and sildenafil is considered as an off-label drug for PAH indication.

Studies show that sildenafil is clinically effective to treat PAH. Sildenafil with supportive treatments, compared to supportive treatments alone, led to significant improvements in exercise capacity (increase in distance of a 6-min walk), haemodynamic outcomes (reduced mean pulmonary arterial pressure and pulmonary vascular resistance), quality of life and improvement of FC in PAH patients [11]. While the United States Food and Drug Administration (US FDA) has approved the use of sildenafil for PAH treatment [8], in Indonesia, pharmaceutical companies have not registered sildenafil with the National Agency of Drug and Food Control for this indication. The drug is merely registered for the indication of erectile dysfunction treatment. As it is an off-label medicine, it cannot be covered under the National Health Insurance.

The PAH association of Indonesia proposed to the Centre for Health Financing and Health Security, Indonesian Ministry of Health, to assess the use of sildenafil for the treatment of PAH and examine its cost-effectiveness if it were to be included in the benefits package. Accordingly, this study aims to assess the cost-effectiveness and budget impact of adopting sildenafil to the benefits package for the indication of PAH, compared to beraprost. The results of this study would be useful for the Indonesian Ministry of Health to consider the use of this off-label medicine and support registration of sildenafil for this indication. Additionally, it would provide evidence for the National Health Insurance to make policy decisions on whether sildenafil should be included in the benefits package for the treatment of PAH.

## Methods

### Economic model

This study is a model-based economic evaluation. A Markov model was used to estimate lifetime costs and health outcomes, comparing sildenafil as the intervention and beraprost as the comparator. The study population included patients who had been diagnosed with PAH based on echocardiography or cardiac catheterization. The inclusion criteria was adult PAH patients (> = 18 years old) who had undergone treatment for at least 3 months. Patients in all functional classes were included. A three-month cycle was used for each FC, which is considered sufficient to capture the treatment effects. The study was performed based on a societal perspective which means that costs incurred by both providers and households were incorporated. All costs and outcomes were discounted at a rate of 3%.

**Fig. 1:**
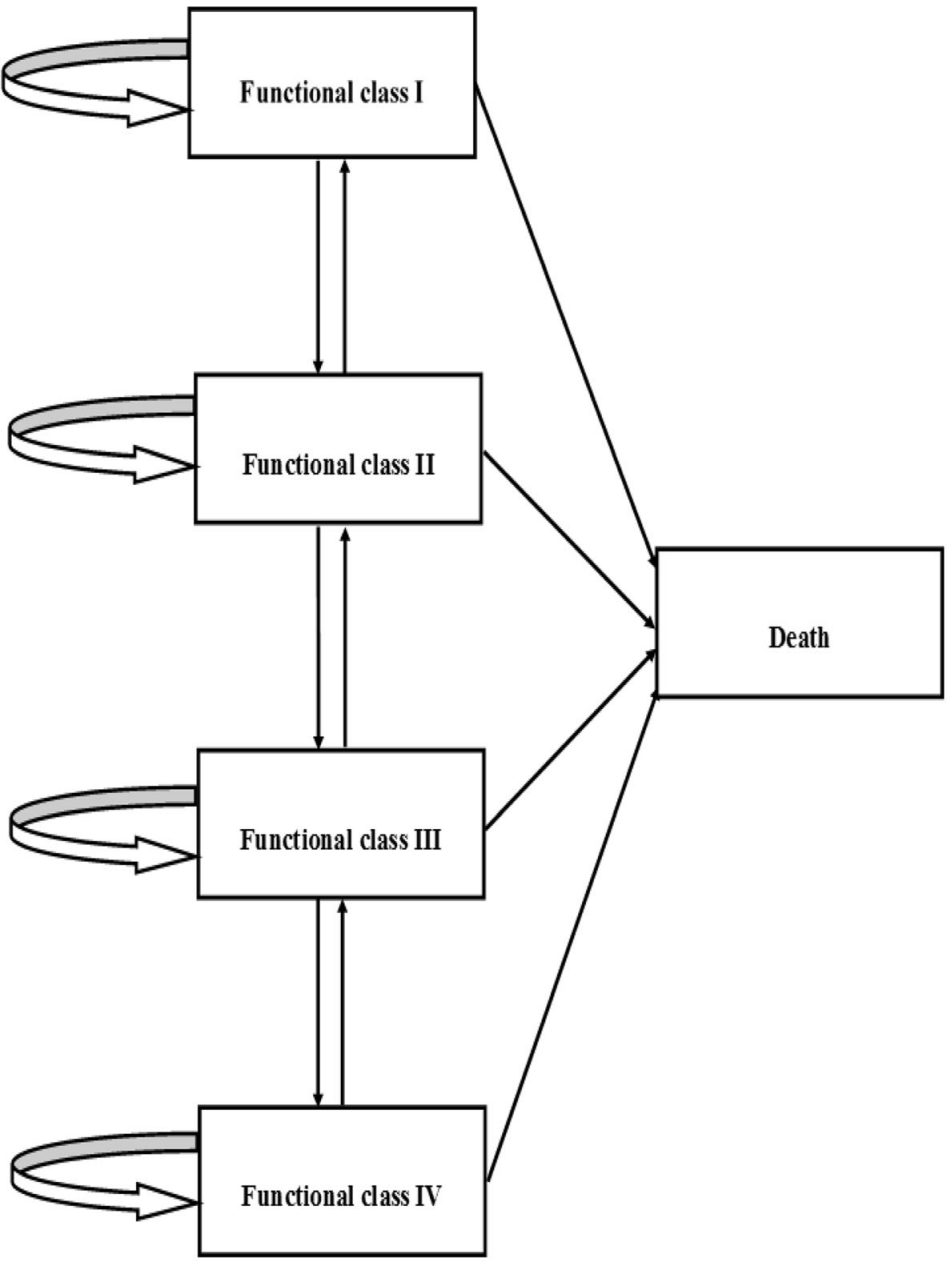
Diagram of the Markov model [12]

Figure 1 illustrates the model based on the health status of the WHO/NYHA functional classification [12]. There are five states in the model, i.e. FC I, FCII, FC III, FC IV and death. The model starts with patients in FC II and FC III since sildenafil is used in those stages according to guidelines for PAH [1]. Patients who receive selective drug therapy can move to other health states and may experience the following states:

- Transitions to a higher FC are defined as FC worsening (e.g. from FC II to FC III)
- Death
- Transitions to a lower FC are defined as FC improvement (e.g. from FC III to FC II)
- Remain in a FC state

### Model input parameters

#### Transitional probabilities

Transitional probability is the probability of patients moving from one state to another state. The probability of switching FC and probability of death in patients receiving pulmonary selective drugs were inputted into the model to simulate disease progression and predict lifetime costs and health outcomes. Relative risk (RR) values of switching FC for patients receiving pulmonary selective drugs compared with standard treatments were applied with the probability of switching FC in patients receiving standard treatments in order to obtain the probability of switching FC in patients receiving pulmonary selective drugs. Transitional probability data and relative risk were obtained from a PAH study conducted by Thongsri W et al., HTA in Thailand (HITAP) [12]. In the study, the transitional probability data and relative risk in patients receiving standard treatments or pulmonary selective drugs were obtained from studies published during 1980–2012 [11, 13–15]. Mortality data for each FC were obtained from a study conducted by Panichwattana W 2010 [16], which was used in the HITAP study [12].

In addition, we also conducted a literature search for studies published during 2012–2015 using Cochrane and MEDLINE database, and identified a systematic review comparing sildenafil with a placebo [17]. The review referred to a PAH study conducted by Galie et al. in 2005 [13], which was also included in the HITAP study.

#### Cost variables

As the perspective of this study is societal, the cost component included direct medical cost and direct non-medical cost. Primary data on direct medical and direct non-medical costs were collected from patients and billing department at two national referral hospitals in Jakarta and Jogjakarta, from July to August 2015. The direct medical cost was taken from hospital billing data, while direct non-medical cost was collected using a structured questionnaire for interviewing the patients. Data collection form and questionnaire developed for collecting direct medical cost and direct non-medical cost are shown in Additional files 1 and 2, respectively.

Direct medical cost included the drug cost, outpatient visit, and hospital admission cost. It was retrieved retrospectively from hospital billing data for a one-year period (2014–2015). There are two types of sildenafil, i.e. generic and originator, which costs USD 0.34 and USD 2.18 per 20 mg tablet, respectively. Beraprost, which is available only at its originator price, costs almost USD 0.33 per 20 mcg.

Direct non-medical cost accounted for travel, consumption, and accommodation expenses when patients visited or were admitted to the hospital. In addition, the opportunity cost of caregivers and the cost of daily supportive devices were calculated. These costs were obtained from a direct interview with 48 patients.

#### Health outcomes

Health outcomes in this study were life years and QALYs. QALYs were calculated by the multiplication of life years and utility values. The utility value was collected from interviews with patients. The same respondents were considered to obtain cost and utility data. We used the EQ-5D-3 L questionnaire, a standardized measure of health status or utility, consisting of questions on 5 dimensions, i.e. mobility, self-care, usual activities, pain/discomfort and anxiety/depression. Each dimension has 3 levels: no problems, some problems, extreme problems. The utility can be calculated by deducting weights (the values of each level in each dimension) from 1 (the value for full health) [18].Thailand’s value set was used to calculate the utility in this study [19]. The instrument was obtained officially from the EuroQol Foundation website.

The values of the parameters and details of primary data collection are shown in Tables 1 and 2, respectively.

**Table 1:** Parameters used in the model

Parameters	Distribution	Mean	SE	References
Probability of switching FC in a patient receiving standard treatment (per 3 months)				
FC I to FC II	Beta	0.127	0.044	[11]
FC II to FC I	Beta	0.125	0.033	
FC II to FC III	Beta	0.127	0.044	
FC III to FC II	Beta	0.125	0.033	
FC III to FC IV	Beta	0.094	0.029	
FC IV to FC III	Beta	0.025	0.023	
Probability of death of PAH patient (standard treatment or pulmonary selective drug)				
Probability of death in FC I	Beta	0.002	0.009	[16]
Probability of death in FC II	Beta	0.013	0.009	
Probability of death in FC III	Beta	0.016	0.011	
Probability of death in FC IV	Beta	0.240	0.065	
Relative risk (RR) of switching FC (compared with standard treatment)				
Sildenafil (FC worsening)^a^	Beta	0.43	0.380	[13]
Sildenafil (FC improvement)^b^	Beta	4.23	2.043	[13, 14]
Beraprost (FC worsening)^a^	Beta	0.10	0.199	[15]
Beraprost (FC improvement)^b^	Beta	0.93	0.612	
Direct medical cost (excluding pulmonary selective drug cost)				
Number of hospital admissions in FC I (per 3 months)	Gamma	0.24	0.03	Patient interviews using questionnaire
Number of hospital admissions in FC II (per 3 months)	Gamma	0.33	0.13	
Number of hospital admissions in FC III (per 3 months)	Gamma	0.20	0.07	
Number of hospital admissions in FC IV (per 3 months)	Gamma	0.50	0.50	
Number of outpatient visits in FC I (per 3 months)	Gamma	5.89	0.48	
Number of outpatient visits in FC II (per 3 months)	Gamma	4.89	0.47	
Number of outpatient visits in FC III (per 3 months)	Gamma	3.00	0.47	
Cost of hospital admission (per admission)	Gamma	808	143	Hospital billing
Cost of outpatient visit (per visit)	Gamma	21	2	
Total direct medical cost of patient in FC I (USD per 3 months)	–	317	–	Patient interviews and hospital billing
Total direct medical cost of patient in FC II (USD per 3 months)	–	368	–	
Total direct medical cost of patient in FC III (USD per 3 months)	–	224	–	
Total direct medical cost of patient in FC IV (USD per 3 months)	–	404	–	
Cost of pulmonary selective drug (USD per 3 months)				
Cost of beraprost (originator price)	–	88	–	Hospital billing
Cost of sildenafil (generic price)	–	92	–	
Direct nonmedical cost (USD per 3 months)				
Direct nonmedical cost in FC I	Gamma	136	27	Patient interviews using questionnaire
Direct nonmedical cost in FC II	Gamma	110	24	
Direct nonmedical cost in FC III	Gamma	158	64	
Direct nonmedical cost in FC IV	Gamma	364	364	
Utility				
Utility of FC I	Beta	0.74	0.04	Patient interviews using questionnaire
Utility of FC II	Beta	0.71	0.04	
Utility of FC III	Beta	0.56	0.03	
Utility of FC IV	Beta	0.51	0.04	

^a^FC worsening means transitions from one FC to higher FC e.g. from FC II to FC III

^b^FC improvement means transitions from one FC to lower FC e.g. from FC III to FC II

USD 1 = IDR 13,830

**Table 2:** Details and sources of primary data collection

Parameters	Components	Sources of data	Number of patients
Direct medical cost	Number of hospital admissions/outpatient visits	Patient interviews	48 (FC I = 18, FC II = 19, FC III = 10, FC IV = 1)
	Hospital admission and outpatient visit costs e.g. other drug cost, laboratory services, etc.	Hospital billing	
Cost of pulmonary selective drugs	Cost of sildenafil and beraprost	Hospital billing	
Direct non-medical cost	Travel, consumption, accommodation expenses, opportunity cost of caregivers, cost of daily supportive devices	Patient interviews	
Utility	–	Patient interviews	

### Data analysis

#### Incremental cost-effectiveness ratio (ICER)

The cost and health outcomes were analysed using the Markov model to calculate incremental cost-effectiveness ratio for each intervention (ICER per QALY gained). ICER was calculated based on the incremental cost of sildenafil and beraprost treatment divided by the incremental QALY gained between these two treatments [20]. Since Indonesia has not established a willingness to pay (WTP) per QALY gained, this study used one times GDP per capita or USD 3109 as the WTP threshold.

#### Uncertainty analysis

A one-way sensitivity analysis was performed to examine the effect of individual parameter uncertainty. A probabilistic sensitivity analysis (PSA) was also conducted using Monte Carlo simulation (1000 times simulation) to examine the effect of all parameter uncertainties and possible values of total costs, health outcomes, and ICER. The results were presented in cost-effectiveness acceptability (CEA) curves, indicating the probability of cost-effective treatment. Additionally, a threshold sensitivity analysis was conducted to determine the cost-effective price of the intervention at the WTP threshold, if it was found to be not cost-effective.

#### Budget impact analysis

Budget impact analysis was conducted to obtain information on how much budget should be provided in the next 5 years. The budget of providing each intervention (sildenafil or beraprost) for PAH patients in FC II and FC III and the incremental budget (the difference in budget of sildenafil compared to beraprost) were calculated. Budget impact was analyzed from a number of PAH patients in FC II and FC III, and cost per patient based on a government perspective. The prevalence and incidence data of those patients were estimated based on international studies as agreed by experts [21]. Eight thousand patients and six hundred and five patients were used as the prevalence and incidence, respectively, in this study. It was estimated that 50% of all patients were PAH patients in FC II and another 50% were PAH patients in FC III. This information would also be useful for relevant agencies to consider whether sildenafil should be included in the benefits package.

## Results

### Health outcomes

Without discounting health outcomes, sildenafil offered more life years saved and more QALYs than beraprost for patients in FC II and FC III (Table 3). Life years gained for the patients in FC II and FC III who received sildenafil was 27.91 and 25.99, respectively. On the other hand, patients who were treated with beraprost gained life years saved of 26.75 for patients in FC II and 23.03 for patients in FC III. In addition, in terms of QALYs, patients who received sildenafil in FC II had 0.9 more QALYs than patients who received beraprost, while patients who received sildenafil in FC III had 2.45 more QALYs than patients who received beraprost.

**Table 3:** Total lifetime cost, life years, and QALYs for PAH patients in FC II and FC III receiving sildenafil and beraprost

	FC II			FC III	
	beraprost	sildenafil		beraprost	sildenafil
Total lifetime cost (USD)	35,863	37,632	30,807		35,059
Life Years					
No discount	26.75	27.91	23.03		25.99
Discount	16.23	16.94	14.10		15.80
QALYs					
No discount	19.66	20.56	16.64		19.09
Discount	11.90	12.47	10.08		11.58

### Cost of policy options

Using a societal perspective and 3% discount rate, the total cost of sildenafil as first-line therapy for patients in FC II and FC III for a lifetime was USD 37,632 and USD 35,059, respectively, while the total cost for beraprost was USD 35,863 and USD 30,807, respectively.

### Incremental cost-effectiveness ratio (ICER)

The incremental cost of providing sildenafil as first-line therapy compared to beraprost was USD 1769 for patients in FC II and USD 4252 for FC III. By discounting the health outcomes, incremental QALYs of sildenafil compared to beraprost was 0.57 for PAH patients in FC II and 1.50 for FC III. ICER per QALY gained for using sildenafil as first-line therapy compared to beraprost for patients in FC II and FC III was USD 3098 and USD 2827, respectively (Table 4).

**Table 4:** Incremental cost-effectiveness ratio per QALY gained

	FC II	FC III
	sildenafil vs beraprost	sildenafil vs beraprost
Incremental Cost (USD)	1769	4252
Incremental QALYs	0.57	1.50
ICER per QALY gained (USD)	3098	2827

Referring to the WTP threshold of USD 3109 per QALY, providing sildenafil for PAH patients in FC II or FC III is cost-effective.

### Uncertainty analysis

**Fig. 2:**
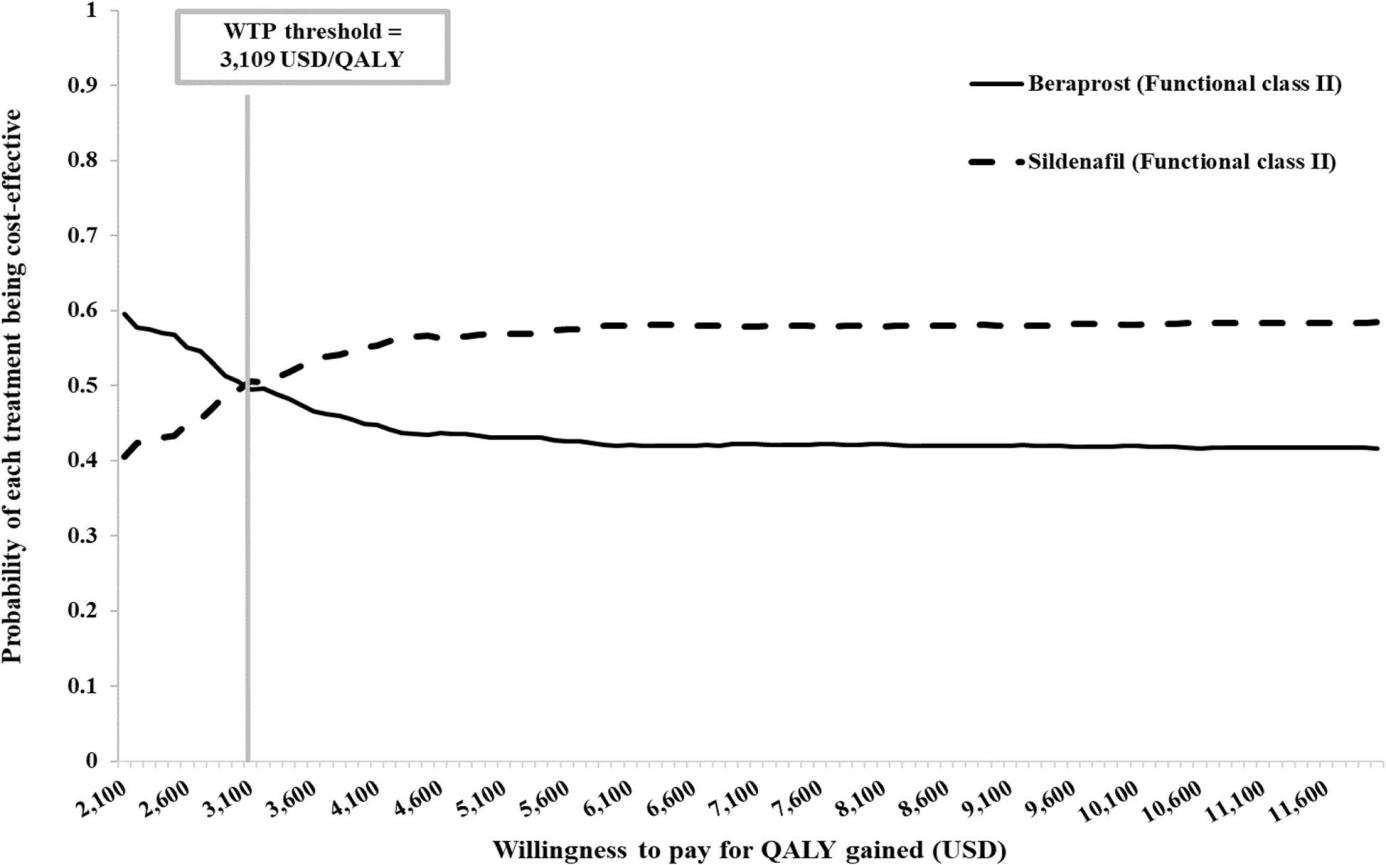
Cost-effectiveness acceptability curve for PAH patients in FC II receiving sildenafil and beraprost

**Fig. 3:**
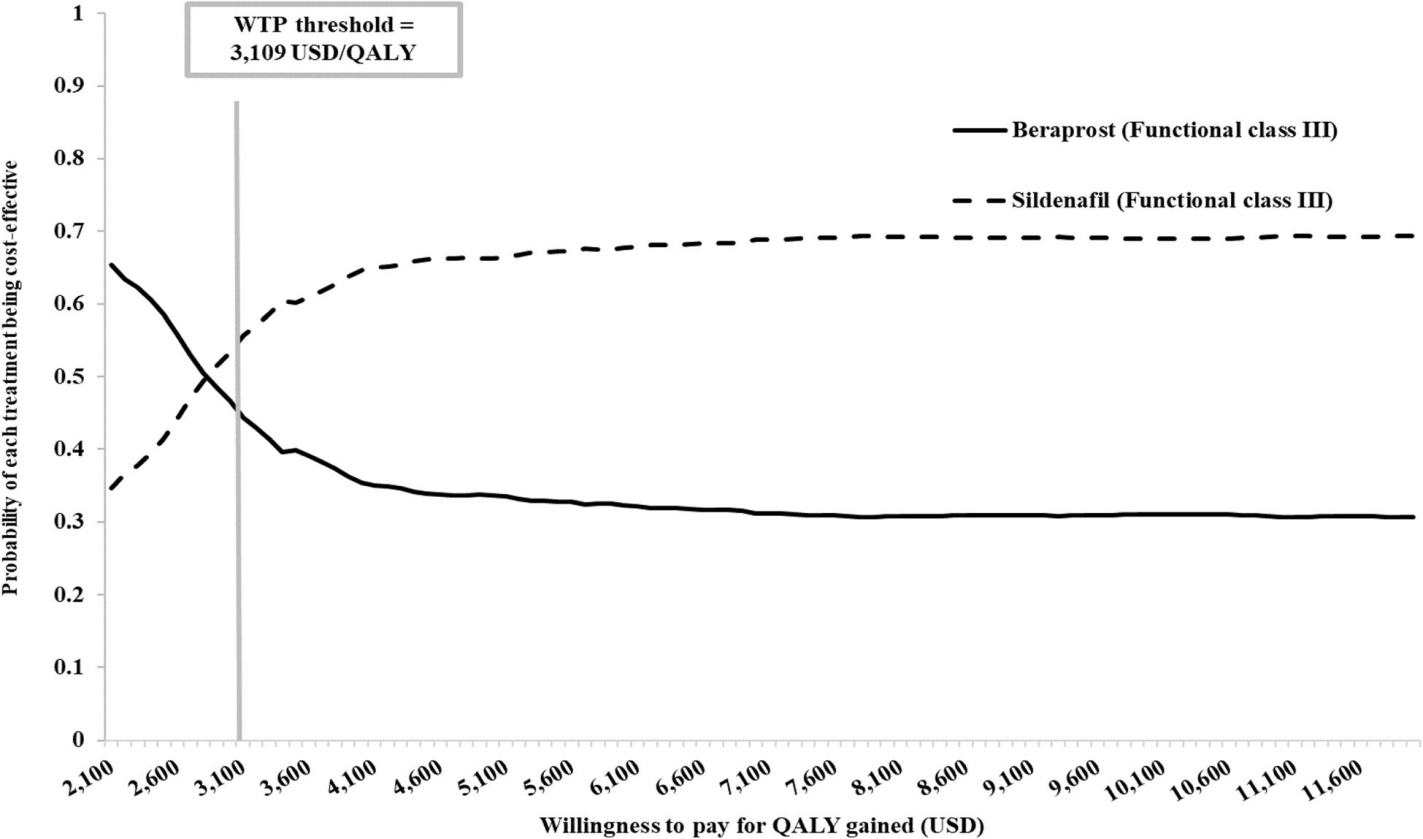
Cost-effectiveness acceptability curve for PAH patients in FC III receiving sildenafil and beraprost

The probabilistic sensitivity analysis is presented in the CEA curve as shown in Figs. 2and 3. At the WTP threshold of USD 3109 per QALY gained, the probability that prescribing beraprost and sildenafil for PAH patients starting in FC II is cost-effective is equal to 50%, while the probability that prescribing beraprost and sildenafil for PAH patients starting in FC III is cost-effective is 44 and 56%, respectively.

In addition, one-way sensitivity analysis was conducted to compare the ICER values between the generic price of sildenafil (USD 0.34 for 20 mg tablet) and its originator price (USD 2.18 for 20 mg tablet). Using the originator price, the ICER value was 20 times higher than the ICER of the generic price for FC II patients, and 8 times higher for FC III patients.

Furthermore, a threshold sensitivity analysis was conducted to obtain information on how much the price of originator sildenafil should be reduced to be cost-effective at the threshold of USD 3109. The results showed that the originator sildenafil could be cost-effective if its price was reduced by 85%.

### Budget impact analysis

By using sildenafil for PAH patients starting in FC II and FC III, the government will spend USD 38.2 million and USD 35.4 million, respectively. The incremental budget of using sildenafil compared to beraprost for PAH patients starting in FC II and FC III was USD 436,775 and USD 3.6 million, respectively (0.08% of Indonesia’s healthcare budget which was USD 4.8 billion in 2015). Table 5 indicates the estimated total budget for 5 years.

**Table 5:** Budget impact for PAH patients in FC II and FC III receiving beraprost and sildenafil^a^

Year	FC II		FC III	
	beraprost	sildenafil	beraprost	sildenafil
1	8.6	8.4	6.6	7.5
2	7.0	6.9	5.9	6.5
3	7.1	7.3	6.2	6.8
4	7.4	7.7	6.4	7.1
5	7.7	8.0	6.6	7.4
Total	37.8	38.2	31.8	35.4

^a^All costs presented in million USD

## Discussion

This study was the first to conduct a full economic evaluation of PAH therapy in Indonesia. We conducted the study using the best available primary data on cost and utility of PAH patients in Indonesia. Cost and utility data were directly obtained from hospital billing and patient interviews. The results demonstrated that compared to beraprost, generic sildenafil is cost-effective for the treatment of PAH patients in FC II and FC III at the WTP threshold. As a result, it could be used as the information for the Healthcare and Social Security Agency (Badan Penyelenggara Jaminan Sosial Kesehatan – BPJS Kesehatan) in order to include sildenafil as the first line treatment for PAH FC II and FC III in the benefits package even though the medicine is not registered for PAH treatment in Indonesia. Meanwhile, the Ministry of Health was proposed to request for Pfizer to register sildenafil for PAH indications, and for local and international pharmaceutical companies to make 20 mg tablets available in Indonesia, instead of only 100 mg tablets, which are used for other indications.

The model estimated that providing sildenafil for PAH patients yield 1–3 additional life years gained (without discounting) as opposed to beraprost. Despite sildenafil’s higher cost compared to beraprost, generic sildenafil could be good value for money in Indonesia.

The Thai study on cost-utility analysis of sildenafil compared to beraprost as the first line therapy for PAH associated with congenital heart disease showed that sildenafil would be the preferable choice for first line treatment [12]. Sildenafil is also included in the National List of Essential Medicines in Thailand for the treatment of PAH patients. Another study was conducted by Garin MC, assessing the cost-utility of sildenafil compared to bosentan, treprostinil, epoprostenol, inhaled iloprost, sitaxentan, and ambrisentan. The economic model of that study showed sildenafil was a cost-effective treatment for PAH with a low price and a net increase in QALYs [22].

Some limitations in our study were the small sample size and unequal number of samples in each FC (FC I: FC II: FC III: FC IV = 18: 19: 10: 1). Only one patient was recruited in FC IV, which might happen because the number of PAH cases in Indonesia is very small and undetected. The unequal number of patients in each FC may lead to high uncertainty in some parameters. To reduce the uncertainty, we conducted a probabilistic sensitivity analysis.

The unavailability of comprehensive data on PAH in Indonesia also limits our study. Relative risk, transitional probability, as well as survival rate of PAH were obtained from other studies [11–16] and experts’ opinions were used to confirm the data (this also applied in another study [12]).

The data was obtained from two hospitals because of limited time and resources. While this may represent only patients from Jakarta and Jogjakarta, these two hospitals were national referred hospitals, and sildenafil treatment was provided only in these hospitals.

The prevalence and incidence data were estimated based on international studies, as agreed by experts. This might not represent the actual number of PAH prevalence in Indonesia since many cases were underdiagnosed, however, it was the best approach to obtain prevalence data.

Another important consideration for this study is that sildenafil has not been registered in the National Drugs Formulary as a pulmonary arterial hypertension therapy, but it is registered for another indication. Therefore, it is necessary to encourage pharmaceutical companies to register sildenafil for PAH in Indonesia.

## Conclusions

Compared to beraprost, sildenafil is a more cost-effective choice for the treatment of PAH patients in functional classes II and III in Indonesia. Although the cost of sildenafil is more expensive than beraprost, generic sildenafil could be included in the benefits package of JKN since it has good value for money. After the completion of the study and consideration of high-level stakeholders and officials, sildenafil went through rapid approval from the pharmaceutical regulatory agency, Badan Pengawas Obat dan Makanan or Badan POM (Indonesia’s National Agency of Food and Drug Control), to be registered for PAH indications in Indonesia. Sildenafil is now part of the national formulary and can be reimbursed through the universal healthcare coverage scheme.

## Additional files

Additional file 1: Data collection form for direct medical cost, Data collection form for collecting direct medical cost from hospital billing data. (DOC 119 kb)

Additional file 2: Questionnaire for direct non-medical cost, Questionnaire for collecting direct non-medical cost from interviewing the patients. (DOC 71 kb)

## Data Availability

Primary data on costs and utility that used and analysed during the current study are available from the Indonesian Health Technology Assessment Committee on reasonable request. The data on direct medical cost was obtained from the databases of Sardjito General Hospital and Harapan Kita Hospital. Public access to the databases is closed. Official request from research team was submitted to those two hospitals to obtain the data and administrative permission was received to access and use it.

## Abbreviations

EQ-5D-3 L: EuroQol 5-dimension 3-level
FC: functional class
FDA: Food and Drug Administration
GDP: Gross domestic product
HITAP: Health Intervention and Technology Assessment Program
HIV: Human immunodeficiency virus
HTA: Health technology assessment
ICER: Incremental cost-effectiveness ratio
IDR: Indonesian Rupiah
JKN: *Jaminan Kesehatan Nasional* (National Health Insurance)
NYHA: New York Heart Association
PAH: Pulmonary arterial hypertension
PSA: Probabilistic sensitivity analysis
QALY: Quality-adjusted life year
QOL: Quality of life
WHO: World Health Organization
WTP: Willingness to pay
