# Supplementary material for: Economic evaluation of sildenafil for the treatment of pulmonary arterial hypertension in Indonesia
Source: BMC Health Serv Res. 2019 Aug 14;19:573. doi: 10.1186/s12913-019-4422-5 (PMC6694473; doi:10.1186/s12913-019-4422-5)
Supplement: Supplementary file 1 — Data collection form for direct medical cost, Data collection form for collecting direct medical cost from hospital billing data. (DOC 119 kb) [file 12913_2019_4422_MOESM1_ESM.doc]

**Data collection form: Direct Medical Cost**

Patient’s phone number:

Inclusion Criteria:

| Adult (≥ 18 years)* |  |
| --- | --- |
| Patients who have been diagnosed with PAH disease based on echocardiography or catheterization* |  |
| WHO functional class I to IV* |  |
| Patients who have undergone the treatment at least 12 weeks in the hospital* |  |

**Put a tick (√) in the box if the patient meets the criterion*

Exclusion Criteria:

- Patients who are referred back to the first hospital
- Patients who died
- Patients who get combination of therapy

PART 1

Patient characteristic:

Q1 Serial number of patient _______________________________

Q2 Medical record number _______________________________

Q3 Age _______________________________

Q4 Sex □ 1. Female □ 2. Male

Q5 Date of first diagnosis □□/□□/□□□□

in any hospital (dd/mm/yyyy)

Q6 Diagnosis tools □ 1. Echocardiography □ 2. Catheterization

Q7 First therapy after diagnosed □ 1. Sildenafil □ 2. Beraprost

in this hospital □ 3. Supportive care □ 4. No treatment

Q8 Starting date of treatment □□/□□/□□□□

(Sildenafil or Beraprost) (dd/mm/yyyy)

in this hospital

Q9 Primary diagnosis □ 1. iPAH □ 2. Heritable PAH

□ 3. CTD PAH □ 4. CHD PAH

□ 5. Other ____________

Q10 ICD-10 code __________________

Q11 Secondary diagnosis _________________ (ICD-10 code______________)

_________________ (ICD-10 code______________)

PART 2

Outpatient expenditure within 1 year (at least 3 months):

*Record 1*

Q12 Date of visit □□/□□/□□□□

(dd/mm/yyyy)

Q13 WHO Functional class □ 1. Class I □ 2. Class II

□ 3. Class III □ 4. Class IV

□ 5. Unidentified

Q14 6-MWT □□□□ meters

(*six-minutes walk test*)

Q15 Current treatment for PAH □ 1. Beraprost □ 2. Sildenafil

□ 3. Iloprost □ 4. Other treatments

Q16 Drug (name of drug) dose for PAH ______X____ a day Total __tablets/ampoules

Drug dose for PAH ______X____ a day Total __tablets/ampoules

Drug dose for PAH ______X____ a day Total __tablets/ampoules

Q17 Expenditure

| Services/items | Expense |
| --- | --- |
| Consultation fees |  |
| PAH drug _______________ |  |
| PAH drug _______________ |  |
| PAH drug ________________ |  |
| Other prescribed medication |  |
| Exercise test |  |
| Laboratory test |  |
| Radiology test |  |
| Echocardiography |  |
| Catheterization |  |
| Registration fee |  |
| Other costs |  |
| **Total expenditure** |  |

*Record 2*

Q18 Date of visit □□/□□/□□□□

(dd/mm/yyyy)

Q19 WHO Functional class □ 1. Class I □ 2. Class II

□ 3. Class III □ 4. Class IV

□ 5. Unidentified

Q20 6-MWT □□□□ meters

(*six-minutes walk test*)

Q21 Current treatment for PAH □ 1. Beraprost □ 2. Sildenafil

□ 3. Iloprost □ 4. Other treatments

Q22 Drug (name of drug) dose for PAH ______X____ a day Total __tablets/ampoules

Drug dose for PAH ______X____ a day Total __tablets/ampoules

Drug dose for PAH ______X____ a day Total __tablets/ampoules

Q23 Expenditure

| Services/items | Expense |
| --- | --- |
| Consultation fees |  |
| PAH drug _______________ |  |
| PAH drug _______________ |  |
| PAH drug ________________ |  |
| Other prescribed medication |  |
| Exercise test |  |
| Laboratory test |  |
| Radiology test |  |
| Echocardiography |  |
| Catheterization |  |
| Registration fee |  |
| Other costs |  |
| **Total expenditure** |  |

*Record 3*

Q24 Date of visit □□/□□/□□□□

(dd/mm/yyyy)

Q25 WHO Functional class □ 1. Class I □ 2. Class II

□ 3. Class III □ 4. Class IV

□ 5. Unidentified

Q26 6-MWT □□□□ meters

(*six-minutes walk test*)

Q27 Current treatment for PAH □ 1. Beraprost □ 2. Sildenafil

□ 3. Iloprost □ 4. Other treatments

Q28 Drug (name of drug) dose for PAH ______X____ a day Total __tablets/ampoules

Drug dose for PAH ______X____ a day Total __tablets/ampoules

Drug dose for PAH ______X____ a day Total __tablets/ampoules

Q29 Expenditure

| Services/items | Expense |
| --- | --- |
| Consultation fees |  |
| PAH drug _______________ |  |
| PAH drug _______________ |  |
| PAH drug ________________ |  |
| Other prescribed medication |  |
| Exercise test |  |
| Laboratory test |  |
| Radiology test |  |
| Echocardiography |  |
| Catheterization |  |
| Registration fee |  |
| Other costs |  |
| **Total expenditure** |  |

*Record 4*

Q30 Date of visit □□/□□/□□□□

(dd/mm/yyyy)

Q31 WHO Functional class □ 1. Class I □ 2. Class II

□ 3. Class III □ 4. Class IV

□ 5. Unidentified

Q32 6-MWT □□□□ meters

(*six-minutes walk test*)

Q33 Current treatment for PAH □ 1. Beraprost □ 2. Sildenafil

□ 3. Iloprost □ 4. Other treatments

Q34 Drug (name of drug) dose for PAH ______X____ a day Total __tablets/ampoules

Drug dose for PAH ______X____ a day Total __tablets/ampoules

Drug dose for PAH ______X____ a day Total __tablets/ampoules

Q35 Expenditure

| Services/items | Expense |
| --- | --- |
| Consultation fees |  |
| PAH drug _______________ |  |
| PAH drug _______________ |  |
| PAH drug ________________ |  |
| Other prescribed medication |  |
| Exercise test |  |
| Laboratory test |  |
| Radiology test |  |
| Echocardiography |  |
| Catheterization |  |
| Registration fee |  |
| Other costs |  |
| **Total expenditure** |  |

PART 3

Inpatient expenditure within 1 year:

*Record 1*

Q36 Date of admission □□/□□/□□□□

(dd/mm/yyyy)

Q37 Date of discharge □□/□□/□□□□

(dd/mm/yyyy)

Q38 WHO Functional class □ 1. Class III □ 2. Class IV

Q39 Current treatment for PAH □ 1. Beraprost □ 2. Sildenafil

During hospitalization □ 3. Iloprost □ 4. Other treatments

and after discharge from hospital

(can be chosen more than one)

Q40 Drug dose for PAH ______X____ a day Total __tablets/ampoules

Drug dose for PAH ______X____ a day Total __tablets/ampoules

Drug dose for PAH ______X____ a day Total __tablets/ampoules

Q41 Expenditure during hospitalization

| Services/items | Expense |
| --- | --- |
| Consultation fees |  |
| PAH drug (during hospitalization and after discharge from hospital) _______________ |  |
| PAH drug (during hospitalization and after discharge from hospital) _______________ |  |
| PAH drug (during hospitalization and after discharge from hospital) ________________ |  |
| Other prescribed medication |  |
| Room fee |  |
| Exercise test |  |
| Laboratory test |  |
| Radiology test |  |
| Echocardiography |  |
| Catheterization |  |
| Paramedic service fee |  |
| Registration fee |  |
| Other costs |  |
| **Total expenditure** |  |

*Record 2*

Q42 Date of admission □□/□□/□□□□

(dd/mm/yyyy)

Q43 Date of discharge □□/□□/□□□□

(dd/mm/yyyy)

Q44 WHO Functional class □ 1. Class III □ 2. Class IV

Q45 Current treatment for PAH □ 1. Beraprost □ 2. Sildenafil

During hospitalization □ 3. Iloprost □ 4. Other treatments

and after discharge from hospital

(can be chosen more than one)

Q46 Drug dose for PAH ______X____ a day Total __tablets/ampoules

Drug dose for PAH ______X____ a day Total __tablets/ampoules

Drug dose for PAH ______X____ a day Total __tablets/ampoules

Q47 Expenditure during hospitalization

| Services/items | Expense |
| --- | --- |
| Consultation fees |  |
| PAH drug (during hospitalization and after discharge from hospital) _______________ |  |
| PAH drug (during hospitalization and after discharge from hospital) _______________ |  |
| PAH drug (during hospitalization and after discharge from hospital) ________________ |  |
| Other prescribed medication |  |
| Room fee |  |
| Exercise test |  |
| Laboratory test |  |
| Radiology test |  |
| Echocardiography |  |
| Catheterization |  |
| Paramedic service fee |  |
| Registration fee |  |
| Other costs |  |
| **Total expenditure** |  |
