# Supplementary material for: Economic evaluation of sildenafil for the treatment of pulmonary arterial hypertension in Indonesia
Source: BMC Health Serv Res. 2019 Aug 14;19:573. doi: 10.1186/s12913-019-4422-5 (PMC6694473; doi:10.1186/s12913-019-4422-5)
Supplement: Supplementary file 2 — Questionnaire for direct non-medical cost, Questionnaire for collecting direct non-medical cost from interviewing the patients. (DOC 71 kb) [file 12913_2019_4422_MOESM2_ESM.doc]

**Questionnaire: Direct Non-Medical Cost**

Inclusion Criteria:

| Adult (≥ 18 years)* |  |
| --- | --- |
| Patients who have been diagnosed with PAH disease based on echocardiography or catheterization* |  |
| WHO functional class I to IV* |  |
| Patients who have undergone the treatment at least 12 weeks in the hospital* |  |

**Put a tick (√) in the box if the patient meets the criterion*

Exclusion Criteria:

- Patients who are referred back to the first hospital
- Patients who died
- **Patients who get combination of therapy**

| Participant ID □□□□ | | | |
| --- | --- | --- | --- |
| Interviewer name : | | | |
| *For each question listed below, please check the appropriate response by making ‘√’ inside the checkbox or writing one number/character in each box.* | | | |
| 1. Date of interview | □□/□□/□□□□  (dd/mm/yyyy) | | |
| 1. Place of interview |  | | |
| 1. Age |  | | |
| 1. Gender |  | | |
| 1. Level of education completed | □ 1. No schooling  □ 2. Primary School  □ 3. Junior High School  □ 4. High School  □ 5. College or above | | |
| 1. What is your current treatment?   (can be chosen more than one) | □ 1. Beraprost  □ 2. Iloprost  □ 3. Sildenafil  □ 4. Standard treatment | | |
| 1. When was your first time of getting current treatment? | □□/□□□□  mm/yyyy | | |
| 1. Who is your accompanying person on a visit to the hospital due to this disease? | □ 1. Nobody (go to question number 11)  □ 2. Family/ Relatives/ Friends (go to question number 9) | | |
| 1. Please indicate the number of your accompanying persons on a visit to the hospital. | □□ persons | | |
| 1. Please indicate age of your accompanying persons on a visit to the hospital. | □□ years old  □□ years old  □□ years old | | |
| 1. What is your average time spending on a doctor visit due to this disease (including round trip traveling, waiting, and administrative times)? | □□ hours | | |
| 1. What is the average cost of a whole trip to a doctor visit (including costs incurred for your accompanying persons)? | Travel | Rp. | |
|  | Food | Rp. | |
|  | Accommodation | Rp. | |
| 1. How many visits were made to your doctor for following up treatment in 3 months period? | □□ times in 3 months | | |
| 1. Are you currently employed earning wages or salary, either full-time or part-time? | □ 1. Yes (go to question number 15)  □ 2. No (go to question number 17) | | |
| 1. Please indicate your average income per month. | Rp. | | |
| 1. Which best describes your current employment situation? | □ 1. Full time job  □ 2. Part time job  □ 3. Enterpreneur/ own business | | |
| 1. If you take a sick off, how much potential wages do you expect to lose per day (including income loss for your family/relatives/friends)? | Rp. | | |
| 1. What was the total income of your household last month? | Rp. | | |
| 1. How many family members are there in your household? | □□ persons | | |
| 1. Have you ever been admitted to the hospital due to this illness/disease? | □ 1. Yes (go to question number 21)  □ 2. No (go to question number 25) | | |
| 1. If Yes (from the question number 20), how many times have you been admitted to the hospital per year? | □□ times per year | | |
| 1. What is the average length of stay in the hospital? | □□ days per visit | | |
| 1. How many family/relatives/friends were there to accompany you during the hospitalization? | □□ persons | | |
| 1. Please indicate age of your accompanying persons while you were hospitalized. | □□ years old  □□ years old  □□ years old | | |
| 1. Did you and your family spend money on the following items? If yes, please provide details on each item. | Supporting equipment | | Rp. |
|  | Child or elder-care | | Rp. |
|  | Any adaptation to your house | | Rp. |
| 1. Are there any family/relatives/friends who helps caring for you at home? | □ 1. Yes (go to question number 27)  □ 2. No (go to question number 31) | | |
| 1. How many family/relatives/friends are there to help caring for you at home? | □□ persons | | |
| 1. Please indicate age of persons who help caring for you at home. | □□ years old  □□ years old  □□ years old | | |
| 1. What is the average amount of time your family/relatives/friends spent on helping you per day? | □□ minutes per day | | |
| 1. How often do your family/relatives/friends help you at home? | □□ days per 3 months | | |
| 1. Have you visited or admitted to another hospital or clinic due to this disease in the past year? | □ 1. Yes (go to question number 32)  □ 2. No | | |
| 1. How many times did you visit another hospital or clinic for the treatment of this disease? | □□ times per 3 months | | |
| 1. What is the average cost of a whole trip to a doctor visit (including costs incurred for your accompanying persons)? | Rp. | | |
